# Photo-oscillations in MgZnO/ZnO heterostructures

**DOI:** 10.1038/s41598-022-27091-0

**Published:** 2022-12-28

**Authors:** Jesús Iñarrea

**Affiliations:** 1 Escuela Politécnica Superior, Universidad Carlos III, Leganes, 28911 Madrid, Spain; 2 Unidad Asociada al Instituto de Ciencia de Materiales, CSIC, Cantoblanco, 28049 Madrid, Spain

**Keywords:** Nanoscience and technology, Physics

## Abstract

We theoretically examine the characteristics of microwave-induced magnetoresistance (MIRO) and photovoltage oscillations in MgZno/ZnO heterostructures. We demonstrate that both kind of oscillations, although described with different physical properties, are intimately related sharing the same physical origin. We use the radiation driven electron orbit model showing that the interplay of radiation driven swinging Landau orbits and the scattering processes are at the heart of the oscillations in both scenarios. Thus, our simulations show that all photo-oscillations present the main features of MIRO: they are periodic with the inverse of the magnetic field and the oscillations minima are 1/4 cycle shifted.

## Introduction

Microwave-induced resistance oscillations (MIRO)^1,2^, envisaged by Ryzhii^3,66^ in the 70’s, are one of the most important effects when it comes to radiation-matter interaction in two dimensional electron systems (2DES). These peculiar oscillations present some distinctive features that serve to identified them. For instance, we can highlight, that they are periodic with the inverse of the magnetic field (*B*), the oscillations minima are shifted in 1/4 of the oscillation cycle and their amplitude exhibits a sublinear law with the power radiation (*P*) that at low power values gets linear. Closely related with the MIRO’s power dependence there is another remarkable effect such as zero resistance states, (ZRS). They shows up when *P* is high enough^1^. Despite the fact that a lot of experimental^4–29,67^ and theoretical^30–47^ works have been carried out, the physical origin of MIRO and ZRS remain still unclear.

MIRO were discovered two decades ago in a high mobility GaAs/AlGaAs heterostructure when measuring irradiated magnetoresistance under a vertical magnetic field (*B*) at very low temperatures, $T \sim 1K$. Later on, similar oscillations were discovered when measuring another physical quantity such as photovoltage^48,50,51^. These novel oscillations presented the same peculiar features as MIRO did, suggesting similar, or at least related, physical origin^52,53^. MIRO have been observed in a bunch of different platforms, all of them holding a system of 2D carriers being irradiated from microwaves to terahertz under moderate *B*. These platforms include heterostructures such as, GaAs/AlGaAs^1,2^, MgZnO/ZnO^54^, GeSi/Ge^55^ and more recently hexagonal boron nitride encapsulated graphene^56–59^.

In this article, we present theoretical results on photo-oscillations of irradiated magnetoresistance ($R_{xx}$) and photovoltage using as platform the MgZnO/ZnO heterostructure^54^. This heterostructure is able to host a 2DES reaching a mobility about $1\times 10^{6}$ cm$^{2}$/Vs with the improvements in the growth techniques^54,60^. This makes MgZnO/ZnO a good candidate to observe MIRO^54^. The goal of this work is to present a common microscopic theory explaining the radiation-induced oscillations experimentally observed in both $R_{xx}$ and photovoltage. This theory stems from the previous model of *the radiation-driven electron orbits model*^30,31,43^ which in turn is based on two main effects: the radiation-driven electron orbit motion and the corresponding scattering of electrons with impurities. According to our model the interplay of both effects would be at the heart of MIRO and photovoltage oscillations. Another important point that makes MgZnO/ZnO heterostructures very interesting and unique is that the source of scattering is different with respect to the most commonly used AlGaAs/GaAs platforms. In the latter case the main source of scattering is long-range potential centers such as remote charged impurities. However in the former, short-range potential scattering centers such as alloy impurities and surface roughness are the predominant contributors to disorder. In the present work, we have treated this kind of scattering with a model of a neutral impurity.

## Theoretical model

The radiation-driven electron orbits model is a previously developed theoretical approach to explain both MIRO and ZRS that were observed in irradiated GaAsAl/GaAs heterostructures. One of the main results of this theory is that the Landau orbits are driven harmonically by radiation and the corresponding guiding center describes harmonic and classical trajectories on the 2D system. Accordingly, the interplay of this driven-harmonic motion and scattering with sample disorder are at the core of photo-oscillations. Previous to irradiating the sample, electrons interact via scattering with the system disorder giving rise to resistance. In principle, the scattering is performed randomly in any direction leading to no net effect. Nevertheless, if there exists a DC electric field on the 2D sample, that can either be externally applied or built-in^48^, a definite direction is determined that, on average, will be followed by the electron when interacting with scatterers. In each scattering jump the scattered electron advances an average distance, $\Delta X_{0}$ along the DC field direction^49^. Thus, a certain current shows up that can be measured in terms of magnetoresistance $R_{xx}$^1,2^ or photovoltage^48^.

The scattering scenario in the dark is deeply altered when the sample is illuminated because the Landau orbits oscillate^30,31,43^. This novel situation can be experimentally observed via $R_{xx}$^1,2^ or, more recently, via photovoltage^48,54^. Now, under radiation, the advanced distance or spatial shift, due to scattering, turns into a harmonic function according to the radiation-driven electron orbit model^30,31,43^,

1 $$\begin{aligned} \Delta X=\Delta X_{0}-A \sin \left( 2\pi \frac{w}{w_{c}}\right) \end{aligned}$$

where *w* and $w_{c}$ are the radiation and cyclotron frequencies respectively and *A* is the oscillation amplitude,

2 $$\begin{aligned} A=\frac{e^{- \gamma \tau /2} e E_{o}}{m^{*}\sqrt{(w_{c}^{2}-w^{2})^{2}+\gamma ^{4}}} \end{aligned}$$

where $E_{0}$ is the radiation electric field amplitude. According to Eq. (1), the radiation-driven Landau states, perform a swinging motion where the electrons interact with the lattice ions resulting in a damping process. The latter is phenomenologically introduced through the $\gamma$ damping term^30,31,43^.

If we now focus on the irradiated $R_{xx}$, we have to calculate first the corresponding conductivity ${\sigma _{xx}}$ following a semiclassical Boltzmann approach^61–63^,

3 $$\begin{aligned} \sigma _{xx}=2e^{2} \int _{0}^{\infty } dE \rho _{i}(E) (\Delta X)^{2}W_{I}\left( -\frac{df(E)}{dE} \right) \end{aligned}$$

being *E* the energy, $\rho _{i}(E)$ the density of initial Landau states and $W_{I}$ is the scattering rate of electrons with sample disorder. According to the Fermi’s golden rule: $W_{I}=N_{i}\frac{2\pi }{\hbar }|<\phi _{f}|V_{r}|\phi _{i}>|^{2}\delta (E_{i}-E_{f})$, where $N_{i}$ is the number of impurities, $\phi _{i}$ and $\phi _{f}$ are the wave functions corresponding to the initial and final Landau states respectively and $V_{r}$ is the disorder scattering potential. $E_{i}$ and $E_{f}$ stand for the initial and final energies. The $V_{r}$ matrix element is given by^61,63^:

4 $$\begin{aligned} |<\phi _{f}|V_{r}|\phi _{i}>|^{2}=\sum _{q}|V_{q}|^{2}|I_{i,f}|^{2} \end{aligned}$$

and the term $I_{i,f}$^61,63^,

5 $$\begin{aligned} I_{i,f}=\int \phi _{f}(x-X^{'}_{0}) exp(i q_{x} x)\phi _{i}(x-X_{0}) dx \end{aligned}$$

where $X_{0}$ and $X^{'}_{0}$ are the guiding centers of the initial and final Landau states respectively and $q_{x}$ the *x*-component of $\overrightarrow{q}$, the electron momentum change after the scattering event.

In the MgZno/ZnO system the main source of disorder and scattering is no longer long-range Coulomb potential centers such as remote charged impurities in AlGaAs/GaAs heterostructures. Now, short-range potential disorder is the main source of scattering. The heterointerface between MgZnO and ZnO takes in most of the disorder due to the Mg atoms that diffuse into the ZnO inversion layer. Thus, to calculate $W_{I}$ we have considered a simple model of a 2D neutral impurity^64^ (Mg atoms) based on a circular barrier of radius *a*. This constant potential is given by: $V_{r}=V_{0}$ if $r\le a$ and $V_{r}=0$ if $r> a$. Thus, $V_{0}$ plays the role of the scattering potential. In the calculation of $W_{I}$ the Fourier transform $V(|\overrightarrow{q}|)$ of the potential $V_{r}$, needs to be obtained and accordingly is given by^64^,

6 $$\begin{aligned} V(|\overrightarrow{q}|)=\pi a^{2}V_{0}\frac{2J_{1}(|\overrightarrow{q}| a)}{|\overrightarrow{q}| a S} \end{aligned}$$

where $J_{1}$ is the first order Bessel function and *S* is the sample surface. Now we consider that $|\overrightarrow{q}|$ is small and thus *V*(*q*) no longer depends on *q* and the scattering becomes isotropic. Then $V(|\overrightarrow{q}|)$ takes the form, $V(|\overrightarrow{q}|)\simeq \pi a^{2}V_{0}/S$. In our simulations we have used for *a* the effective Bhor radius^64^ that in the case of ZnO is of the order of 2 nm. For $V_{0}$ we have used for a neutral impurity an estimate of^61,65^ $V_{0}\sim 50$ meV. Finally, $R_{xx}$ is calculated according to the usual tensorial relations, $R_{xx}=\frac{\sigma _{xx}}{\sigma _{xx}^{2}+\sigma _{xy}^{2}}$, where $\sigma _{xy}\simeq \frac{n_{i} e}{B}$, $n_{i}$ being the electrons density, and *e* the electron charge. Then, and according to Eq. (3), the distance $\Delta X$ is direct responsible of the rise of MIRO when measuring $R_{xx}$.

**Figure 1:**
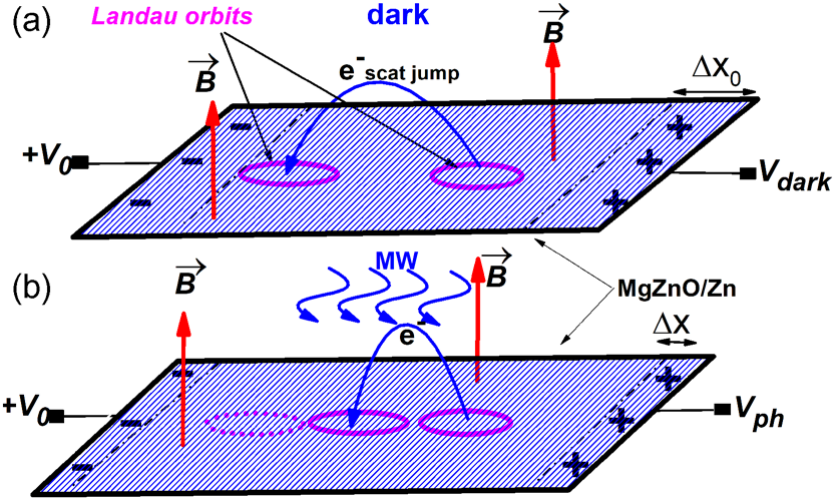
Schematic diagram of the microscopic model of dark voltage and photovoltage in MgZnO/ZnO heterostructures. The scattering direction is determined by the external voltage $+V_{0}$. (**a**) In the dark case the scattered electron mostly jump, between Landau orbits, in the direction of the positive voltage. As a result two lines of opposite charges build up at facing sides of the sample. (**b**) With radiation, the scattering jump distance changes due to the swinging motion of driven Landau orbits giving rise to radiation-induced photovoltage oscillations. The case of a shorter distance jump regarding the dark case is shown.

**Figure 2:**
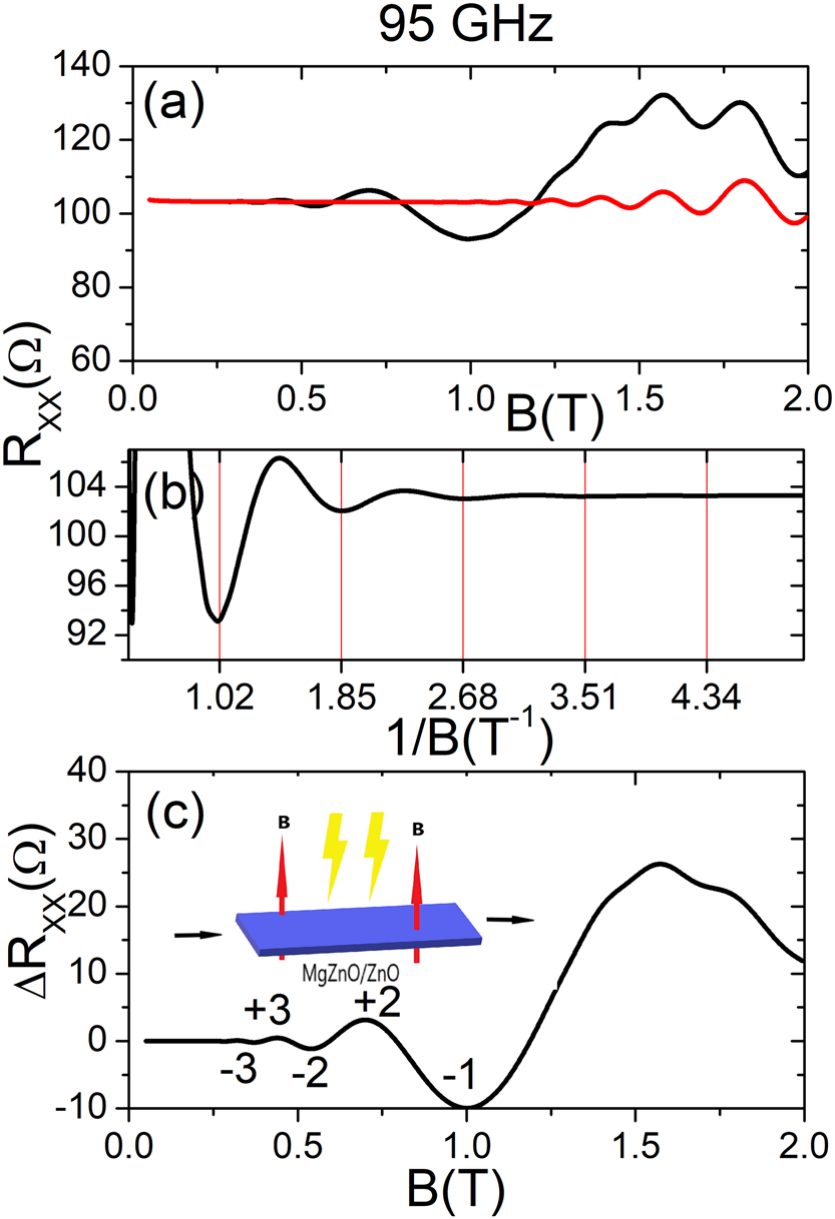
(**a**) Calculated irradiated (black curve) and dark (red curve) $R_{xx}$ vs *B*. The radiation frequency is 95 GHz and $T=1.0$ K. SdH oscillations are observed in both curves and in the irradiated one MIRO. (**b**) Irradiated $R_{xx}$ vs the inverse of *B*. The corresponding curve is perfectly periodic. (**c**) Calculated MIRO amplitude $\Delta R_{xx}$ vs *B*. The labels in the figure correspond to the extrema positions (maxima and minima). The inset shows the basic experimental setup.

**Figure 3:**
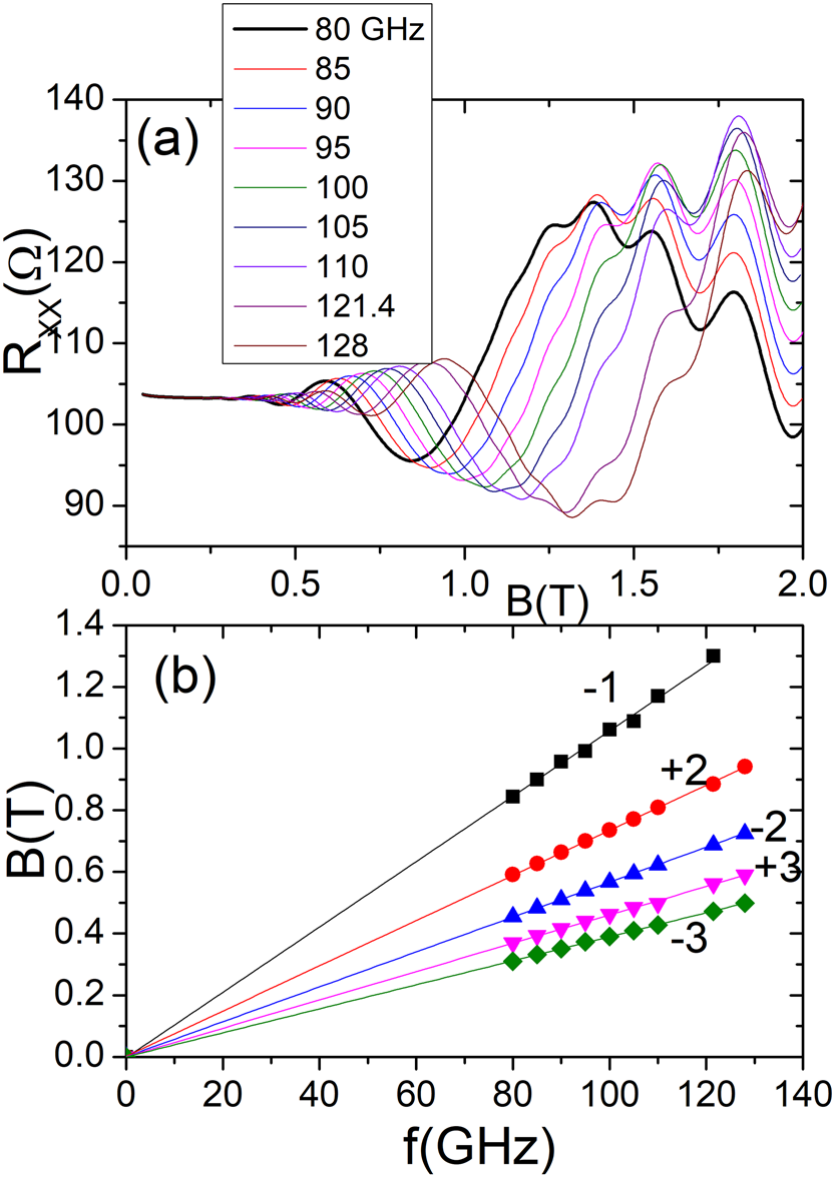
Frequency dependence of irradiated $R_{xx}$ vs *B*. In the upper panel we exhibit irradiated $R_{xx}$ vs *B* for nine different frequencies. Frequencies run from 80 to 128 GHz. Oscillations displace to higher *B* as frequency increases. In the lower panel the five groups of points correspond to the extrema positions labelled in Fig. 2. They show the dependence of extrema positions with *B*. The fits show that this dependence follow a straight line in all cases.

The joint effect of radiation-driven Landau states and impurity scattering as origin of photo-oscillations can be revealed by measuring photovoltage instead of irradiated $R_{xx}$^59^. As we have indicated above, in order to obtain a net scattering we need a predominant direction along which the scattering jump takes place. This direction is determined by a DC electric field acting on the sample. In the case of $R_{xx}$ this DC field is externally applied^1,2^. Nevertheless, in the case of photovoltage it can be either built-in^48^ due, for instance to the presence in the sample of metallic connection pads, or externally applied^56^. To theoretically study photovoltage in MgZno/ZnO systems we have applied a model previously used for encapsulated graphene^59^. In it, one of the squared sample edges is connected to an external positive DC voltage, $+V_{0}$ and then a definite scattering direction is determined in the sample (see Fig. 1). As a result two lines of opposite charge rise at facing sides. The lines width is of the order of the scattering spatial shift between Landau states^30,31,43^ $\Delta X_{0}$. Then, a voltage drop, $V_{dark}$, is created along the sample and can be experimentally measured. An expression for $V_{dark}$ can be obtained, from basic electrostatics,

7 $$\begin{aligned} V_{dark}=\frac{n_{2D} e \Delta X_{0}}{2\pi \epsilon } \end{aligned}$$

where *e* is the electron charge, $\epsilon$ is the ZnO permittivity and $n_{2D}$ is the 2D charge density on the lines. $n_{2D}$ can be expressed in function of the density of states per unit area around the Fermi energy $D(E_{F})$, $n_{2D}(E_{F})= 2 D(E_{F}) \Delta E_{F}$, where $\Delta E_{F}$ is an energy interval around the Fermi energy. Thus, $D(E_{F})$ accounts for the SdH oscillations that show up in the photovoltage. Under irradiation $\Delta X_{0}$ turns into a harmonic function^44^ $\Delta X=\Delta X_{0}-A \sin \left( 2\pi \frac{w}{w_{c}}\right)$ and instead of $V_{dark}$ there is a photovoltage, $V_{ph}$ given by an expression similar to $V_{dark}$ but with $\Delta X$ instead of $\Delta X_{0}$.

**Figure 4:**
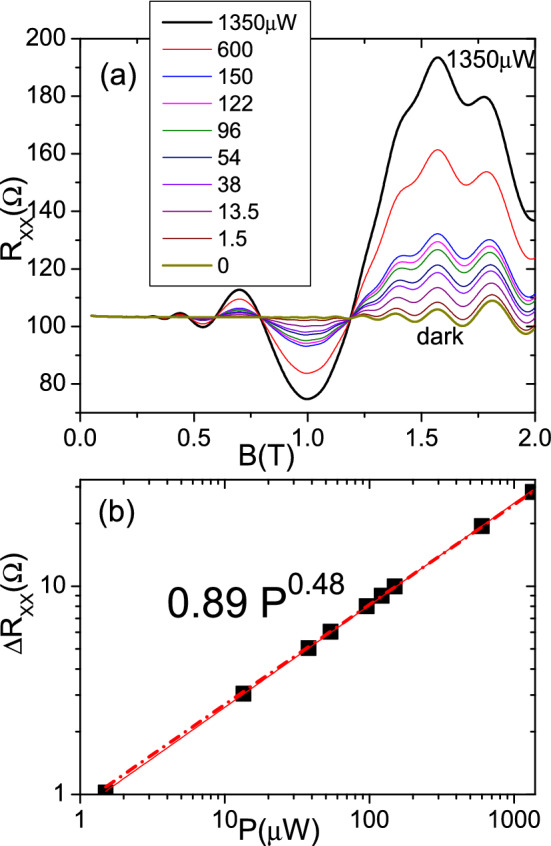
In the upper panel we present the radiation power dependence of irradiated $R_{xx}$ vs *B*. Ten curves are exhibited corresponding to ten different power values ranging from the dark case to 1350 $\upmu$W. As *P* rises the oscillations amplitude increases too. On the other hand *P* does not alter the oscillations position that keep constant as *P* increases. In the lower panel we presene the irradiated $R_{xx}$ amplitude vs *P*. $T=1$ K.

**Figure 5:**
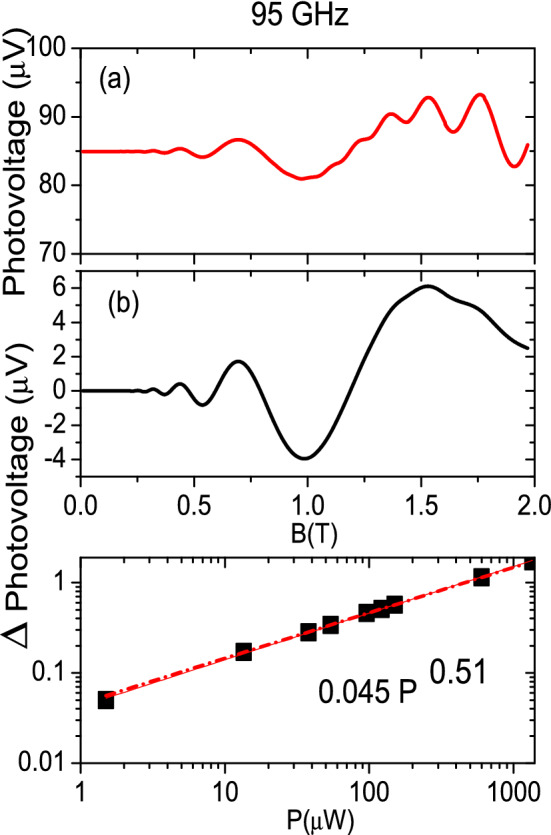
Frequency dependence of the calculated photovoltage vs *B*, showing four frequencies in the THz range: 0.7, 1, 1.5 and 2.0 THz. As the frequency rises the number of oscillations increases but the oscillations amplitude lowers. $T=1$ K.

## Results

In Fig. 2 upper panel we exhibit two curves of $R_{xx}$ vs *B* for MgZnO/ZnO. The black curve corresponds to irradiated $R_{xx}$ and the red curve to the dark case. The radiation frequency is 95 GHz and $T=1.0$ K. In both curves we can observe SdH oscillations and in the irradiated also MIRO, as occurs in other semiconductor platforms. The main photo-oscillations shows up at higher *B* regarding AlGaAs/GaAs due to the bigger effective mass. In our simulations for MgZnO/ZnO we have used $m^{*}=0.35 m_{e}$ where $m_{e}$ is the bare electron mass^54^. In contrast, for AlGaAs/GaAs the usual effective is around $m^{*}=0.067m_{e}$ and the oscillations show up at smaller *B*. For the electron density we have used $n_{e}= 5 \times 10^{11}\,\, {\text {cm}}^{-2}$ and a mobility of $1\times 10^{5}\,\, {\text {cm}}^{2}$/Vs. These are similar values as the ones of the experiments^54^. In the middle panel we represent irradiated $R_{xx}$ vs the inverse of *B*. The corresponding curve is perfectly periodic, as expected fulfilling one of the distinctive MIRO features. The vertical lines at the minima positions act as a help to check the oscillations periodicity with 1/*B*. In the lower one we exhibit the oscillations amplitude $\Delta R_{xx}$ vs *B*, so that he extrema positions can be more easily identified. The labels in the figure correspond to the extrema (maxima and minima). The oscillations minima show the shift of 1/4 in the oscillation cycle, which is another peculiar MIRO feature. Thus, their position varies as $w/w_{c}=1/4 +j$, *j* being a positive integer. The inset shows the basic experimental setup. The calculated results are in qualitatively agreement with experiment^54^.

In Fig. 3 we plot the radiation frequency dependence of irradiated magnetoresistance in MgZnO/ZnO. In the upper panel we present irradiated $R_{xx}$ vs *B* for nine different frequencies from 80 to 128 GHz. All the curves are 1/4 cycle shifted irrespective of the frequency. We observe that MIRO displace to higher *B* as frequency increases, increasing as well the number of oscillations. The extrema change their positions in the *B* axis according to a definite dependence; for the minima according to $w/w_{c}=1/4 +j$, *j* and for the maxima, $w/w_{c}=3/4 +j$, *j*. This dependence is revealed in the lower panel where we represent *B* versus radiation frequency for five cases corresponding to the extrema labelled in Fig. 2 lower panel. We have carried out fits on every group of points obtaining very clear straight lines, as expected according to the two previous formula that relate *w* and $w_{c}$. The variation of extrema positions with *B* according to a straight line is another genuine characteristic of MIRO and has been experimentally observed in previous semiconductor platforms. Again the calculated results are in qualitatively good agreement with the experimental ones^54^.

Figure 4 shows the power dependence of irradiated magnetoresistance in MgZnO/ZnO. In the upper panel we present irradiated $R_{xx}$ vs *B* for ten different power values ranging from 0 to 1350 $\upmu$W. As the power increases the MIRO amplitude rises too following a square root law: $\Delta R_{xx} \propto P^{0.5}$. The *P* increase does not affect the oscillations position that keep constant. The power Law dependence is exhibited in the lower panel where we present $\Delta R_{xx}$ amplitude vs *P*. We fit the calculated points obtaining a square root dependence (dashed red curve) of $\Delta R_{xx}$ with *P*: $\Delta R_{xx}= 0.89 P^{0.48}$. Despite the controversy between linear and square root dependence, we have to admit that a good number of experiments show a mixed behaviour between linear and sublinear. The most recent experiments with encapsulated monolayer graphene^56^, show a linear dependence at low power and as the latter increases, the dependence becomes sublinear.

We now focus on the photovoltage results. Thus, in the upper panel of Fig. 5 we exhibit photovoltage vs *B* for a frequency of 95 GHz and $T=1$ K. The curve is qualitatively very similar to the one of irradiated $R_{xx}$ in Fig. 2a. In the middle panel we exhibit photovoltage amplitude vs *B*. Both curves in (a) and (b) present the main characteristics of MIRO that were already obtained when calculating magnetoresistance: photovoltage turns out to be periodic with 1/*B* (not shown in the figure) and minima positions are 1/4 cycle shifted. The extrema positions coincide with the ones obtained in irradiated $R_{xx}$. Finally in the lower one we observe that photovoltage follows a square root law when it comes to radiation power dependence.

## Conclusions

Summing up, we have theoretically studied the microwave-induced resistance oscillations and photo oscillations experimentally found in MgZnO/ZnO heterostructures. We have used the radiation-driven electron orbit model to depict a common microscopic model for both kind of oscillations. We have come to the conclusion that the interplay between the radiation-driven Landau orbits that perform harmonic trajectories and the interaction with the sample disorder are at the origin of both photo-oscillations. Thus both show the main distinctive characteristics of previous MIRO. They are periodic with the inverse of the magnetic field and they are 1/4 cycle shifted. The calculated results both, magnetoresitance and photovoltage are in qualitative good agreement with experiments^54^ except in the part of power dependence where experiments shows a linear behavior at low radiation power.

## Data Availability

All data generated or analysed during this study are included in this published article.
